# The Therapeutic Effect of Adipose-Derived Mesenchymal Stem Cells for Radiation-Induced Bladder Injury

**DOI:** 10.1155/2016/3679047

**Published:** 2016-03-08

**Authors:** Xuefeng Qiu, Shiwei Zhang, Xiaozhi Zhao, Kai Fu, Hongqian Guo

**Affiliations:** ^1^Department of Urology, Affiliated Drum Tower Hospital, Nanjing University, Nanjing 210008, China; ^2^Institute of Urology, Nanjing University, Nanjing 210093, China

## Abstract

This study was designed to investigate the protective effect of adipose derived mesenchymal stem cells (AdMSCs) against radiation-induced bladder injury (RIBI). Female rats were divided into 4 groups: (a) controls, consisting of nontreated rats; (b) radiation-treated rats; (c) radiation-treated rats receiving AdMSCs; and (d) radiation-treated rats receiving AdMSCs conditioned medium. AdMSCs or AdMSCs conditioned medium was injected into the muscular layer of bladder 24 h after radiation. Twelve weeks after radiation, urinary bladder tissue was collected for histological assessment and enzyme-linked immunosorbent assay (ELISA) after metabolic cage investigation. At the 1 w, 4 w, and 8 w time points following cells injection, 3 randomly selected rats in RC group and AdMSCs group were sacrificed to track injected AdMSCs. Metabolic cage investigation revealed that AdMSCs showed protective effect for radiation-induced bladder dysfunction. The histological and ELISA results indicated that the fibrosis and inflammation within the bladder were ameliorated by AdMSCs. AdMSCs conditioned medium showed similar effects in preventing radiation-induced bladder dysfunction. In addition, histological data indicated a time-dependent decrease in the number of AdMSCs in the bladder following injection. AdMSCs prevented radiation induced bladder dysfunction and histological changes. Paracrine effect might be involved in the protective effects of AdMSCs for RIBI.

## 1. Introduction

Pelvic radiotherapy is one of the standard therapies for a wide variety of tumors localized in the rectum, uterus/cervix, prostate, and urinary bladder. In spite of recent advances that improve range and direction of radiation, radiotherapy is unavoidably associated with normal tissue side effects. One of the organs that are frequently affected by pelvic radiotherapy is the urinary bladder [1, 2]. Radiation-induced bladder injury (RIBI) usually occurs in three phases. A reversible early response starts during the treatment and resolves within several weeks after radiotherapy. After a symptom-free latent phase, an irreversible and progressive late response occurs [1, 3]. The mechanisms of radiation-induced bladder injury (RIBI), however, are little known and the effective treatment strategies for RIBI are still limited [4, 5].

Mesenchymal stem cells (MSCs), firstly identified in bone marrow, have been demonstrated to be pluripotent cells with great potential to differentiate into various mesodermal lineages [6]. Additionally, MSCs are capable of secreting different types of cytokines, which have trophic effects on cytoprotection, cell survival, and immunomodulation [7]. Imamura and his colleagues have demonstrated that implantation of bone marrow derived MSCs (BmMSCs) had beneficial effects in improving bladder function in a rat model of RIBI [8], suggesting the potential use of MSCs as an alternative treatment strategy for RIBI.

Adipose tissue represents an abundant and accessible source of mesenchymal stem cells (AdMSCs) [9]. Several comparison studies have shown that AdMSCs are similar in cell surface expression profiles, differentiation potential, and therapeutic efficacy to BmMSCs [10]. Most importantly, sufficient number of AdMSCs for clinical application could be obtained with minimal side effects under local anesthesia [11]. Our recent study indicated that injection of freshly isolated autologous adipose-derived stromal vascular fraction (SVF) enhanced recovery of erectile function in a rat model of cavernous nerve injury [12]. Administration of AdMSCs has been shown to improve bladder function and result in preservation of bladder microstructure in a rat model of bladder dysfunction associated with hyperlipidemia [13], diabetes mellitus [14], and bladder outflow obstruction [15]. However, it still remains unknown whether administration of AdMSCs has beneficial effect on improving bladder function of RIBI.

This study was therefore designed to explore the effects of AdMSCs in improving bladder function in a rat model of RIBI. Also, the possible mechanism involved in AdMSCs improving bladder function of RIBI was explored. To our knowledge, this is the first study to explore the feasibility of using AdMSCs to rescue radiation-induced bladder injury.

## 2. Materials and Methods

### 2.1. Animal Groups and Study Design

Sixty-two female Sprague-Dawley rats (10 weeks old) were used in our study. X-ray was delivered to the bladder area of the rats to induce RIBI animal model. Radiated rats were randomly divided into three groups: radiation control group (RC, *n* = 18), AdMSCs treated group (AdMSCs, *n* = 18), and AdMSCs conditioned medium treated group (Medium, *n* = 8). Twenty-four hours after radiation, AdMSCs or AdMSCs conditioned medium was administered to AdMSCs group and Medium group, respectively. At the 1 w, 4 w, and 8 w time points after cells injection, 3 randomly selected rats in RC group and AdMSCs group were sacrificed and urinary bladder samples were harvested to detect AdMSCs in bladder tissue. Twelve weeks after injection, the remaining rats were sacrificed and bladders were harvested for molecular analysis after undergoing bladder function test. Ten rats without radiation were set as the normal control (NC, *n* = 8). Ten additional rats were used for isolation and culture of AdMSCs. All animal procedures were approved by the institutional animal care and use committee at Nanjing University.

### 2.2. Establishment of RIBI Animal Model

For animals, the rats were anesthetized with ketamine (100 mg/kg) and midazolam (5 mg/kg). A lead shield with a 25 mm × 15 mm window was used to limit the radiation to the bladder according to our previously used protocol [16, 17]. A single dosage of 20 Gy [18] was delivered with a linear accelerator (Siemens, 6-MV X-ray, 2 Gy/min).

### 2.3. Preparation of AdMSCs and AdMSCs Conditioned Medium

AdMSCs were isolated and cultured as described previously [19]. Briefly, adipose tissue was incubated with 0.075% type I collagenase (Sigma-Aldrich, St. Louis, MO) for 1 h at 37°C. After centrifuging at 220 g for 10 min, the remaining cells were suspended in Dulbecco's modified Eagle's medium (DMEM) supplemented with 10% fetal bovine serum (FBS) and plated at a density of 1 × 10^6^ cells in a 10 cm dish. AdMSCs were passed under the same conditions through not more than five passages before being used for assays.

To prepare AdMSCs conditioned medium, 1 × 10^6^ AdMSCs were seeded in FBS supplemented DMEM medium on 6-well dish. The culture medium was changed into serum-free DMED medium. Twenty-four hours later, the medium was collected and centrifuged at 3,000 ×g for 5 min. The supernatant was collected and concentrated.

### 2.4. Flow Cytometry

Briefly, 1 × 10^5^ AdMSCs were harvested and suspended in 500 *μ*L PBS, incubated with anti-CD29 (Bio-Legend, San Diego, CA, USA), anti-CD44 (Bio-Legend), anti-CD45 (Millipore Corporation, Billerica, MA, USA), or anti-CD31 (Santa Cruz Biotechnology, Santa Cruz, CA) for 2 hours at room temperature in darkness. After washing with PBS 3 times, AdMSCs were analyzed using FACSCalibur (Becton Dickinson, USA). AdMSCs incubated with fluorescence-conjugated IgG isotypes were used as negative controls.

### 2.5. Administration of AdMSCs or AdMSCs Conditioned Medium

After anesthetization, a skin incision was made to expose the bladder and either 800 *μ*L serum-free DMED medium containing 1 × 10^6^ or 800 *μ*L concentrated AdMSCs conditioned medium was evenly injected into the muscular layer of bladder with a 25 G needle according to our previously described protocol [13, 14, 17]. AdMSCs were labeled with the thymidine analog 5-ethynyl-2-deoxyuridine (EdU, Invitrogen, Carlsbad, CA, USA) before injection. After treatment, the incision was closed in 2 layers.

### 2.6. Metabolic Cage Evaluation

According to a previously described protocol [20], all rats were acclimatized in the metabolic cage for 24 h for the measurement of 24 h urine output. All rats were treated equally during the same period after which the urine was collected in a container containing 5 mL of liquid paraffin to prevent evaporation.

### 2.7. Histological Evaluation

Middle parts of bladder tissues were fixed in 4% paraformaldehyde in PBS at 4°C overnight, after which the tissue was transferred to 30% sucrose in PBS at 4°C overnight. The tissues were embedded in Optimal Cutting Temperature (OCT) and stored at −80°C.

For immunofluorescence staining, sections were incubated with 3% goat serum for 30 min at room temperature. Bladder sections were then incubated with primary antibodies overnight at 4°C, followed by Alex594-conjugated goat anti-rabbit IgG (Invitrogen, Carlsbad, CA, USA, 1 : 200) for 1 h at room temperature. The primary antibodies used in the present study was rabbit anti-CD31 (Santa Cruz Biotechnology, Santa Cruz, CA, USA, 1 : 200). EdU staining was performed as described before.

For Masson's trichrome staining, the slides were stained using commercial available kits (Jiancheng, Nanjing, China) following the protocols provided by the manufacturer.

### 2.8. Image Analysis and Quantification

For tissue specimens, data were averaged on 6 sections from each bladder. Images were captured on a Nikon microscope with a Spot RT color digital camera, and digital histomorphometric analysis was performed using Image-Pro Plus 6.0 software (Media Cybernetics, Silver Spring, MD, USA).

### 2.9. Enzyme-Linked Immunosorbent Assay (ELISA)

Protein concentration of tumor necrosis factor-*α* (TNF-*α*), transforming growth factor-*β*1 (TGF-*β*1), and interleukin-1*β* (IL-1*β*) in bladder tissue protein lysates was determined using a commercial available ELISA system (R&D system, Minneapolis, MN, USA). Results were expressed in pg/g protein based on standard recombinant protein curves.

### 2.10. Statistics

Results are expressed as mean ± standard deviation and data were analyzed by one-way analysis of variance (ANOVA) to compare among multiple groups followed by post hoc comparisons with the least significant difference test using Prism 4 (GraphPad Software, San Diego, CA, USA). A *P* value < 0.05 was considered statistically significant.

## 3. Results

### 3.1. Characterization of Cultured AdMSCs

**Figure 1:**
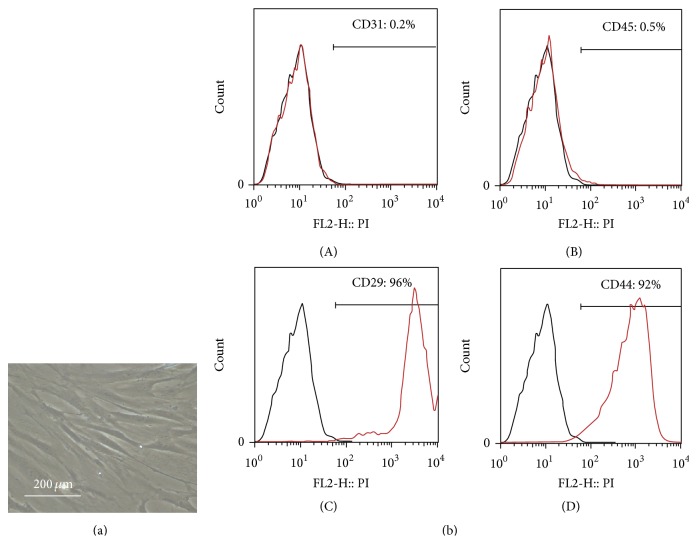
Morphology, flow cytometric analysis of AdMSCs. (a) Phase-contrast image of AdMSCs. (b) Flow cytometric analysis showed that cultured AdMSCs expressed CD29, CD44, but not CD31 or CD45. Black lines indicate isotype control while red lines indicate positively stained cells of each marker.

The cultured AdMSCs demonstrated a spindle-shaped morphology (Figure 1(a)). FACS analysis demonstrated that cultured cells expressed CD29 and CD44, but not CD31 and CD45 (Figure 1(b)), indicating that cultured cells were of mesenchymal origin with high purity.

### 3.2. Tracking Injected BM-MSCs in Bladder Tissue

**Figure 2:**
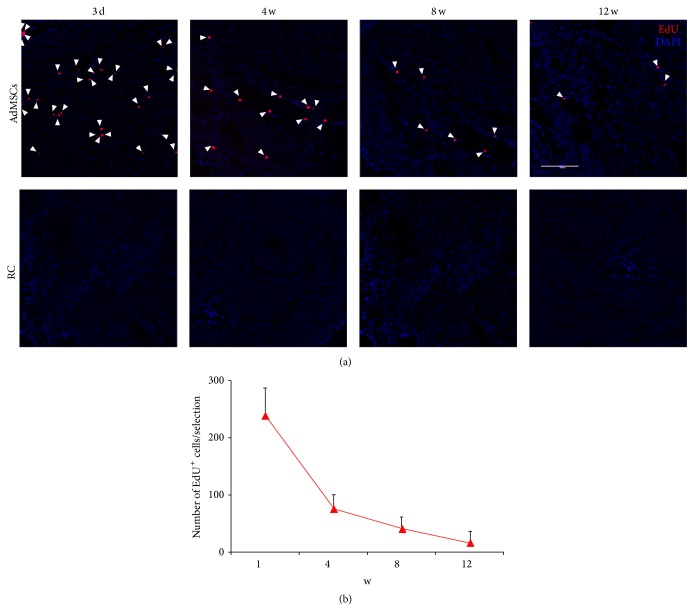
Tracking of injected AdMSCs in bladder tissue. (a) Up: representative images of EdU positive cells within the bladder wall at different time points after cell injection. The white bar indicates 200 *μ*m. Bottom: results of the number of EdU positive cells within the bladder wall at different time points after cell injection.

AdMSCs were labeled with EdU before bladder injection. Histological data indicated a time-dependent decrease in the number of AdMSCs in the bladder following injection. Twelve weeks after injection, very limited number of AdMSCs could be observed within the bladder (Figure 2).

### 3.3. AdMSCs or AdMSCs Conditioned Medium Improves Bladder Function of Diabetic Rats

**Figure 3:**
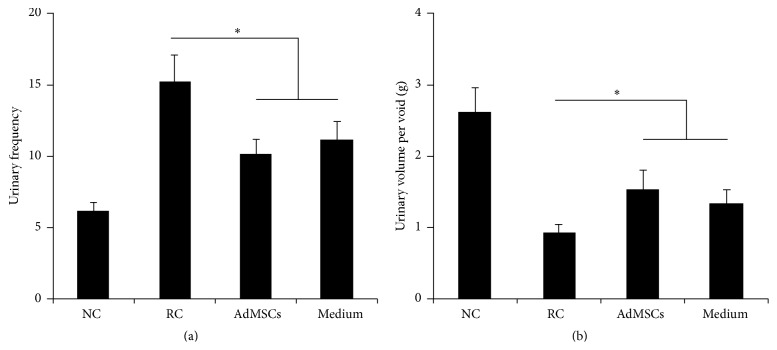
Results of metabolic cage investigation. ^*∗*^ *P* < 0.05 compared with the RC group. There is no significant difference between AdMSCs group and Medium group.

As shown in Figure 3, urinary bladder dysfunction in RC group was indicated by significantly increased urinary frequency and decreased urinary volume per voiding. Partial but significant recovery of bladder function was observed in the treated groups. This is reflected by significantly decreased urinary frequency and increased urine volume.

### 3.4. AdMSCs or AdMSCs Conditioned Medium Reduced Fibrosis and Inflammation within the Bladder Wall

**Figure 4:**
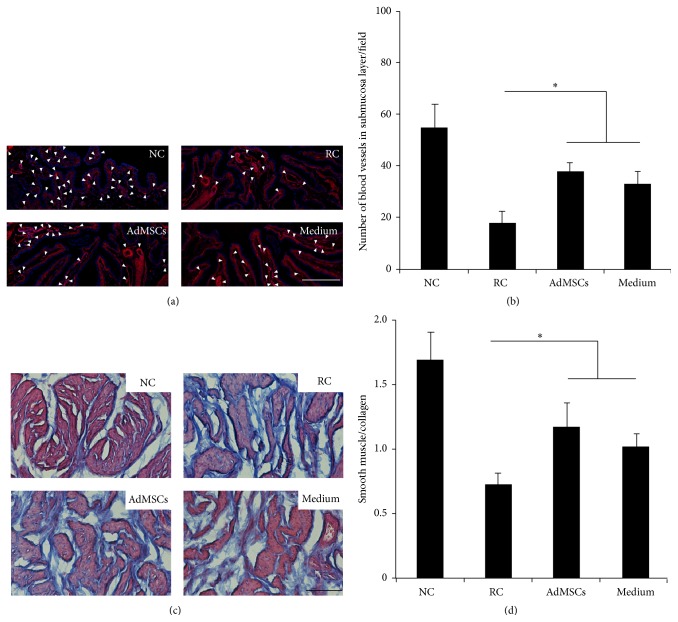
Histological analysis of bladder tissue. (a) Representative images of blood vessels in the submucosa layer. The white arrows indicate blood vessels while the white bar indicates 200 *μ*m. (b) Results of the number of blood vessels in the submucosa layer of each experimental group. ^*∗*^ *P* < 0.05 compared with RC group. There is no significant difference between AdMSCs group and Medium group. (c) Representative images of Masson's trichrome staining from each group. The black bar indicates 50 *μ*m. (d) Results of the ratio between smooth muscle and collagen within the bladder wall of each experimental group. ^*∗*^ *P* < 0.05 compared with RC group. There is no significant difference between AdMSCs group and Medium group.

**Figure 5:**
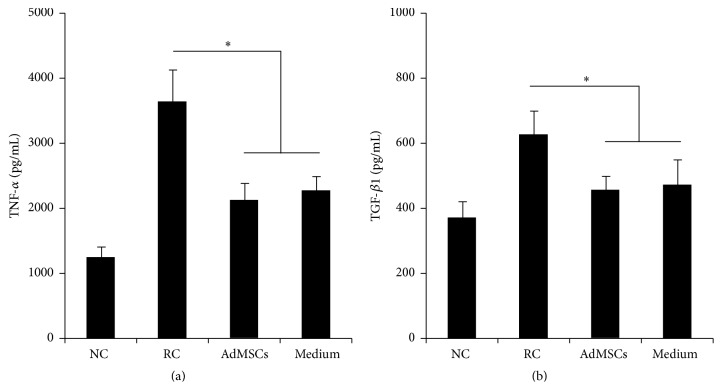
Results of the expressions of TNF-*α* and TGF-*β*1 in the bladder of each group. ^*∗*^ *P* < 0.05 compared with the RC group. There is no significant difference between AdMSCs group and Medium group.

The increased number of blood vessels was observed in the submucosa in treated rats (Figures 4(a)-4(b)). Similarly, bladder administration of AdMSCs or AdMSCs conditioned medium partially but significantly reduced the collagen/muscle ratio in bladder (Figures 4(c)-4(d)). AdMSCs or AdMSCs conditioned medium also altered the levels of immunoregulatory cytokines. AdMSCs or AdMSCs conditioned medium treated rats showed decreased levels of TNF-*α* and TGF-*β*1 (Figure 5). AdMSCs or AdMSCs conditioned medium did not alter the levels of IL-1 *β* (data not shown).

## 4. Discussion

RIRI associated symptoms such as increased urinary frequency, urgency, and dysuria appear early after the treatment and resolve within a few weeks after the end of radiotherapy [18]. The pathology of acute phase of RIBI is primarily believed to be the damage of bladder mucosa and subsequent inflammatory response within the bladder tissue [21]. The underlying pathology of late phase of RIBI is different from that in the early phase. The prominent feature of radiation induced tissue damage is often considered as hypovascular, hypocellular, and hypoxic. The capability to replace normal collagen and cell loss is compromised in hypoxic conditions, which results in tissue breakdown. Fibrosis that occurs partly during repair process can lead to a reduction of bladder capacity [3]. In the present study, a single dose of 20 Gy of X-ray was applied to establish a rat model of RIBI. Our results demonstrated significantly increased collagen deposition (Figure 4) and expressions of inflammatory cytokines 12 weeks after radiation (Figure 5), which suggest fibrosis and inflammation in the bladder tissue. In addition, the number of blood vessels in the submucosa was significantly decreased after radiation. These pathological features were consistent with the previously reported pathological features of RIBI, suggesting the successful establishment of a rat model of RIBI.

AdMSCs have attracted rising interest for regenerative medicine due to sufficient adipose sources and easy isolation [12]. AdMSCs are capable of self-renewal and multidifferentiation. In addition, cytotherapy using AdMSCs may have good proregenerative effects for the injured tissue [10]. It has been demonstrated that AdMSCs were effective in repairing radiation-induced damage on intestine [22], salivary gland [23], or erectile tissue [16] damage. In the present study, we firstly demonstrated the therapeutic effects of AdMSCs for RIBI. Our results indicated that bladder administration of AdMSCs early after radiation process successfully protected against radiation-induced bladder function impairment (Figure 3). In addition, histological results also indicated that radiation-induced inflammation (Figure 5) and fibrosis (Figure 4) were alleviated by the treatment of AdMSCs.

BmMSCs have been demonstrated to be effective in restoring radiation-impaired bladder function [8]. However, the underlying mechanism still remains unclear. In the previous study, the histological results indicated that implanted BmMSCs could be differentiated into smooth muscle cells or nerve cells. Therefore, differentiation of BmMSCs into smooth muscle layers or nerve fibers was proposed as one of the main mechanisms involved in BmMSCs improving bladder function of RIBI [8]. In the present study, we counted the number of EdU labeled AdMSCs at different time points after cell injection. The results revealed that a steep decrease of AdMSCs within the bladder wall occurred from one week to four weeks after injection. The number of AdMSCs in the bladder kept deceasing in a time-dependent way. There were very a limited number of AdMSCs existing within the bladder wall 12 weeks after cell injection (Figure 2). A multitude of studies performed in solid organs showed that more than 80%–90% of grafted cells die within 72 hours after cell transplantation [24–26]. Different mechanisms have been proposed to be involved in the early death of grafted cells, including local oxidative stress, hypoxia, and inflammation [27–29]. Oxidative stress and inflammation are prominent at the early phase of RIBI [3]. Therefore, it is reasonable to speculate that injected AdMSCs died due to the oxidative stress and inflammation in the radiated bladder, suggesting that differentiation of AdMSCs into tissue cells makes limited contribution to the therapeutic effects of AdMSCs for RIBI and some other mechanisms might be involved.

Recently, increasing evidence indicates the role of paracrine effect of AdMSCs in promoting tissue repair. AdMSCs are capable of producing different types of cytokines, which have trophic effects on cytoprotection, cell survival, and immunomodulation [30]. Some studies have demonstrated that even condition medium [30] or cell lysate [31] of AdMSCs had regenerative effects for damaged tissue. In the present study, injection of AdMSCs conditioned medium also resulted in improved bladder function and preserved histology. This data, combined with the limited survival cells within bladder, provided indirect evidence supporting our hypothesis that paracrine pathways of AdMSCs are involved in preventing bladder dysfunction induced by radiation. The histological data indicated that conditioned medium increased the number of blood vessels in the submucosa area and reduced the fibrosis of muscular area. The ELISA data indicated that the expression of TGF-*β*1, a molecular marker of fibrosis [32], and TNF-*α*, a molecular marker of proinflammation [33], was reduced after the treatment of conditioned medium. Taking together, it is not difficult to speculate that the beneficial effect on bladder function observed in our study might be, in part, attributable to the presence of molecules which have endothelial protective, antifibrotic, or anti-inflammation effects.

Our study has some limitations. First, we only assessed some cytokines in the bladder tissues after the treatment. Further studies designed to detect a wider panel of cytokines are needed. Second, although paracrine effect of AdMSCs is shown to be one of the possible mechanisms, it is still unknown what the exact contents are and how these are responsible for the improved function and preserved structure. Further studies aimed at identifying the key factors released by AdMSCs are also needed.

In conclusion, administration of AdMSCs is effective in improving bladder function and preserving bladder microstructure of RIBI. Paracrine effect of AdMSCs is responsible for the functional improvement and structural preservation.
